# Facet theory and the mapping sentence: Evolving philosophy, use, and declarative applications 2nd ed. 2021 edition-Paul M. W. Hackett

**DOI:** 10.3389/fpsyg.2023.1008663

**Published:** 2023-02-28

**Authors:** Ava Gordley-Smith

**Affiliations:** School of Communication, Emerson College, Boston, MA, United States

**Keywords:** facet theory, declarative mapping sentence, mapping sentence, qualitative psychology, social science research, philosophical perspectives, declarative approach

## Introduction

In *Facet theory and the mapping sentence: Evolving philosophy, use, and declarative applications*, Paul M. W. Hackett illustrates Facet Theory's expansive evolution and diverse application. This second edition of the former Facet theory and the mapping sentence: Evolving philosophy, use, and application (2014) is twice as long and exhibits a title amendment with the addition of “declarative.” Whereas, the first edition largely regarded qualitative facet theory, the second edition delves deeply into the development and application of the declarative mapping sentence. The thrust of the work is substantially more philosophical, ethnographic, and qualitative in nature. Hackett's expressive second edition is a compelling read for students, academics, and researchers within the disciplines of the humanities, behavioral sciences, and social sciences.

Hackett methodically illustrates the fundamentals of Facet Theory and mapping sentences whilst journeying the reader through the diverse co-existing utilizations. He encapsulates these dynamic capabilities through descriptive vignettes ranging from studies of avian cognition and behavior to prison officers' occupational stress and the Black Lives Matter movement. He writes, “Facet theory and the mapping sentence offer a potentially constructive philosophical basis for both the understanding of daily existence and inquiries into personal ontologies and mereologies” (p 214). Whilst echoing the instructive crux of the first edition, the second edition gives prominence to the traditional mapping sentence and the declarative mapping sentence. Hackett is also keen to address the mapping sentence's evolved consideration and ability to stand alone as a research tool outside of Facet Theory.

## Discussion

Facet Theory was originally proposed by Louis Guttman as a quantitative research approach for the empirical sciences (Shye, 1999). Hackett's declarative adaptation of the mapping sentence is an evolution of Guttman's work expanding into qualitative branches. Hacket explicates the differences between the traditional mapping sentence and the declarative mapping sentence and the necessity for the declarative adaptation. Hackett's amended mapping sentence, the declarative mapping sentence, provides a reflexive framework that responds to input from broad derivations whilst a traditional mapping sentence holds a stipulated and outcome variable form. The declarative mapping sentence does not impose a structure upon the data but the responsive framework is transportable from one situation and data source to another, reacting to each by allowing alterations. The declarative mapping sentence stimulates cumulative knowledge development, avoiding the imposition of assumptions present in a more rigid design and analysis system, rather, it creates space for knowledge emergence.

An ancillary function to this knowledge emergent space seen in Hackett's declarative mapping sentence is the utilization of one's personal vernacular and the engaged kinship of linguistic determinism. In other words, the declarative mapping sentence requires the researcher to develop their thoughts through their own linguistic systems leading to an intimacy uninhibited by an imposed outcome. This rapport shepherds companionship that is deepened through facet iteration and ultimately leads to a holistic interplay of self and research knowledge emergence. Whilst this symbiotic reflexivity might demand meticulous adjustments, it is this iterative and unconfined linguistic proximity that substantiates the declarative mapping sentence's unique attributes.

One might consider the likeness of this process to therapeutic journaling. Journaling is a commonly known personal writing strategy to express oneself and was made notable as a psychoanalytic Freudian and cognitive Alderian method in the twentieth century (Schneider and Stone, 1998). Journaling exercised in academic or clinical settings may be employed to gain insight through personal reflection, analysis, cooperative dialogue, and feedback (Haertl, 2008). This self-dialogical method expresses a comparable interplay between linguistic determinism, reflexivity, iteration, and knowledge emergence. Similarly to the declarative mapping sentence, a journal imposes no structure upon the data, it exists blankly, only structured by the facets and connective phraseology of one's mental maps. With continued engagement it becomes an iterative process of reflection, maturing into a body of work that improves more comprehensively with time. The process of reading one's own words develops direction and creates a clarifying distance whilst allowing for deeper insight formation; a personal didactic. True to form, Hackett eloquently journals his own experiences unveiling the declarative mapping sentence's notable ability to form an intimacy and companionship between the researcher and their research.

## Conclusion

The process of creating and employing a mapping sentence is a body of work on its own merits. Notable Facet Theorist David Canter wrote, “Facet theory is still only slightly recognized by psychologists and other social sciences. Even when it is accepted, it is only partially understood” (Canter, 1985). Although spotted acknowledgments of Canter's sentiment may exist, Hackett's demonstrated extensive and diverse contributions to Facet Theory assist its expansion, awareness, and necessity. And whilst critics dismiss the Facet Theory and the mapping sentence as overly laborious, Hackett's compelling exposition for the enlightenment found through the application offers a persuasive contention for the recognition of their multitudinous benefits. Hackett's *Facet theory and the mapping sentence: Evolving philosophy, use, and declarative applications* is a philosophical didactic that masterfully traces the evolution and use of Facet Theory.

## Author contributions

The author confirms being the sole contributor of this work and has approved it for publication.
